# Mr. Syer's Case of Haematemesis

**Published:** 1805-03-01

**Authors:** John Syer

**Affiliations:** Bishopsgate Street


### To Dr. BRADLEY.

Dear Sir,

THE following case of Hæmatemesis having recently occurred, and not recollecting any detail of this species of hæmorrhage in the usual routine of medical communications, I take the liberty of requesting you to give it insertion in the next number of the Medical Journal.

I am, &c.

*Bishopsgate Street, Jan.* 15, 1805. JOHN SYER.

---

Perhaps there is no subject of human pathology at present better understood, or wherein cases are more successfully treated, than hæmorrhage. This certainly arises from our late acquaintance with the physiology of the vascular system, and a more minute knowledge of the properties of the blood. It might therefore appear superfluous, a priori, to accumulate further materials for the improvement of practice; still it must be acknowledged, that the event of every hæmorrhage will be more or less precarious, according to its situation; and therefore the medical treatment of internal hæmorrhage cannot be altogether uninteresting.

I was called, on the 12th of November last, to a young lady, of a full habit, but regular as to her modes of life, who had voided, by the act of vomiting, about 12 ounces of dark coloured blood, mixed with a small portion of bile and the usual secretion of the stomach. About ten or twelve hours previous to the accident, she had complained of pain, not very acute, in the epigastric region, and a good deal of nausea, which she attributed to some article of food that had occasioned much restlessness during the night. On first seeing her, the countenance was pale, the circulation somewhat hurried; there was a degree of universal coldness to the touch, a continuance of nausea in a slight degree, an unpleasant brackish taste in the mouth, and the patient was anticipating a return of the hæmorrhage. About a fortnight before this period she had been somewhat indisposed by a sense of languor, and failure of appetite to eat, fancied herself bilious, but could not afford any satisfactory information that could explain the cause of this hæmorrhage.

Before

Before I had an opportunity to send any thing for her relief, the hæmorrhage had recurred; but she was directed to take a draught of the infus. rosæ compos. with two scruples of magnesia vitriolata and five drops of tinct. opii every four hours, and the following morning a draught of manna and a larger quantity of magnes. vitriol.; to persist in the horizontal posture, and to abstain from vinous liquors and animal food. Very early on the morning of the 13th, she experienced a return of hæmorrhage, and had lost full 14 ounces of blood, of the same dark complexion, which coagulated as it was voided; a violent degree of vertigo and distressing sickness preceded each attack. She was now much exhausted; the purgative operated rather copiously, and there appeared with the fæces a considerable quantity of a dark chocolate coloured fluid of a highly offensive smell, intermixed with portions of coagulated blood. The pulse was from 120 to 130, accompanied with a throbbing sensation, and she had strong palpitation of the heart. She became rather alarmed, and was very irritable; every little noise or motion in the room harrassed her, and she had truly the aspect of a patient suffering from severe uterine hæmorrhage. The skin was rather clammy, and the pupil of the eye considerably dilated, which gave to that organ a glassy lustre. She was desired to persevere in the same medicines, and passed the remainder of the day and nearly the whole of the ensuing night without any alarming symptom, or return of sickness, but was seldom free from an aching pain about the stomach, and a considerable degree of dyspnœa.

On the 14th, about six o'clock in the morning, the hæmorrhage returned, (preceded by extreme sickness) to the amount of 12 ounces. The family were now much disheartened, and with difficulty concealed their sentiments from the patient; they were therefore solicitous of further advice, and a physician of considerable eminence was called in, who regarded the case as a critical one, and substituted oleaginous medicines with a small quantity of tinct. Rhæi, in lieu of the infusion of roses; at the same time directing recourse to be had to her former medicines, (which were never rejected) provided the present composition should create any nausea. Her diet consisted purely of farinaceous substances and milk; and extreme quietness was enjoined. The pulse still indicated a disposition to relapse, but she remained free from every other precursory symptom; she had violent head-ach, and the palpiration tation of the heart gradually subsided. The bowels were rather too constipated, and the oleaginous medicines were repeated, with a double portion of the vin. Rhæi, which was still insufficient to obviate constipation, and therefore a mixture of ol. Ricini was directed. The physician discontinued his visits, and the patient was now consigned to my care. The same plans were persisted in, and she gradually recovered from the effects of the hæmorrhage, which, however, had left her very debilitated.

During her convalescence, the appetite for food was voracious, but the stomach easily disordered, and any little indulgence above the usual quantity was succeeded by pain and flatulence. The pulse gradually returned to its usual standard, but the head-ach and nervous affections alternately troubled her. She was not suffered to change her position for nine days after the last attack of hæmorrhage; and to this and other regulations in diet and medicine, which were punctually followed, might, in a great measure, be ascribed her exemption from further danger. The only symptom that tended to retard her perfect recovery, was an almost insufferable constipated state of the bowels, notwithstanding the exhibition of very powerful cathartics. No degree of œdema, or other characteristic of weakness, has supervened.

The indications of cure in hæmorrhage from the stomach, probably, do not differ much from what would be suggested in hæmorrhage from other parts of the body. The general principle of diminishing the force of the circulating powers must necessarily be attended to; the mind must be kept tranquil; and the qualities of those substances we administer, be particularly attended to. Another point may be worthy of observation, namely, the propriety of exciting the peristaltic motion of the intestines by such medicines as are not calculated to excite too powerfully the action of their muscular coats. Probably, the best mode of accomplishing this may be through the medium of injections; but I have no intention of establishing any position from the result of a solitary case, and shall leave any further comments upon it to your more literary Correspondents.

